# Underlying disease risk among patients with fatigue: a population-based cohort study in primary care

**DOI:** 10.3399/BJGP.2024.0093

**Published:** 2024-12-10

**Authors:** Becky White, Nadine Zakkak, Cristina Renzi, Meena Rafiq, Arturo Gonzalez-Izquierdo, Spiros Denaxas, Brian D Nicholson, Georgios Lyratzopoulos, Matthew E Barclay

**Affiliations:** Epidemiology of Cancer Healthcare and Outcomes (ECHO) Research Group, Department of Behavioural Science and Health, Institute of Epidemiology & Health Care, University College London, London, UK.; Department of Behavioural Science and Health, Institute of Epidemiology & Health Care, University College London, London; Cancer Intelligence, Cancer Research UK, London, UK.; ECHO Research Group, Department of Behavioural Science and Health, Institute of Epidemiology & Health Care, University College London, London, UK; associate professor, Faculty of Medicine, University Vita-Salute San Raffaele, Milan, Italy.; ECHO Research Group, Department of Behavioural Science and Health, Institute of Epidemiology & Health Care, University College London, London, UK; Department of General Practice and Primary Care, Centre for Cancer Research, University of Melbourne, Melbourne, Australia.; Centre for Health Data Science, Institute of Applied Health Research, University of Birmingham, Birmingham; Institute of Health Informatics (IHI), University College London, London, UK.; IHI, University College London, London; British Heart Foundation Data Science Centre, London, UK.; Nuffield Department of Primary Care Health Sciences, University of Oxford, Oxford, UK.; Epidemiology of Cancer Healthcare and Outcomes (ECHO) Research Group, Department of Behavioural Science and Health, Institute of Epidemiology & Health Care, University College London, London, UK.; Epidemiology of Cancer Healthcare and Outcomes (ECHO) Research Group, Department of Behavioural Science and Health, Institute of Epidemiology & Health Care, University College London, London, UK.

**Keywords:** diagnosis, cancer, fatigue, primary health care, prodromal symptoms, cohort studies

## Abstract

**Background:**

Presenting to primary care with fatigue is associated with a wide range of conditions, including cancer, although their relative likelihood is unknown.

**Aim:**

To quantify associations between new-onset fatigue presentation and subsequent diagnosis of various diseases, including cancer.

**Design and setting:**

A cohort study of patients presenting in English primary care with new-onset fatigue during 2007–2017 (the fatigue group) compared with patients who presented without fatigue (the non-fatigue group), using Clinical Practice Research Datalink data linked to hospital episodes and national cancer registration data.

**Method:**

The excess short-term incidence of 237 diseases in patients who presented with fatigue compared with those who did not present with fatigue is described. Disease-specific 12-month risk by sex was modelled and the age-adjusted risk calculated.

**Results:**

The study included 304 914 people in the fatigue group and 423 671 in the non-fatigue group. In total, 127 of 237 diseases studied were more common in men who presented with fatigue than in men who did not, and 151 were more common in women who presented with fatigue. Diseases that were most strongly associated with fatigue included: depression; respiratory tract infections; insomnia and sleep disturbances; and hypo/hyperthyroidism (women only). By age 80 years, cancer was the third most common disease and had the fourth highest absolute excess risk in men who presented with fatigue (fatigue group: 7.01%, 95% confidence interval [CI] = 6.54 to 7.51; non-fatigue group: 3.36%, 95% CI = 3.08 to 3.67; absolute excess risk 3.65%). In women, cancer remained relatively infrequent; by age 80 years it had the thirteenth highest excess risk in patients who presented with fatigue.

**Conclusion:**

This study ranked the likelihood of possible diagnoses in patients who presented with fatigue, to inform diagnostic guidelines and doctors’ decisions. Age-specific findings support recommendations to prioritise cancer investigation in older men (aged ≥70 years) with fatigue, but not in women at any age, based solely on the presence of fatigue.

## Introduction

Fatigue is a common presenting symptom in primary care and the principal complaint in around one in 15 consultations.^1^^–^^3^ The assessment of patients presenting with new-onset fatigue aims to rule out serious disease such as cancer by considering the patient’s age, sex, medical history, other presenting signs/symptoms,^4^ and first-line primary care diagnostic tests.^5^

The current authors’ previous research quantified the short-term risk of cancer in patients with fatigue.^6^^,^^7^ However, fatigue is a non-specific symptom with low positive predictive value for many conditions. Existing UK diagnostic guidelines include autoimmune diseases, chronic fatigue syndrome, chronic infections, post-viral fatigue, coeliac disease, depression, diabetes, heart disease, hypothyroidism, and vitamin deficiency.^4^^,^^5^ Such recommendations are based largely on case–control studies examining prodromal features of diseases considered in isolation. No cohort study has quantified the risk of multiple diagnostic outcomes in patients presenting with fatigue in a population-based setting, which could support doctors’ referral decisions and testing strategies.

This study aimed to contextualise short-term disease risk in patients presenting with new-onset fatigue and identify diseases with the strongest associations with fatigue. The risk of cancer was compared to other diseases, as contextualising the relative likelihood of this time-sensitive diagnosis could help prioritise referral and investigation strategies.

## Method

### Study design

A cohort study of patients with a record of fatigue in primary care in England was conducted, using electronic health records (EHRs) from a large population-based dataset — Clinical Practice Research Datalink (CPRD) GOLD.^8^ Diagnoses recorded in secondary care from 1 April 1997 to 31 October 2020 were identified through linkage with Hospital Episodes Statistics (HES) Admitted Patient Care (APC) data.^9^ Cancers recorded from 1 January 1995 to 31 December 2018 were identified through linkage with the national cancer registry.^10^

**Table:** How this fits in

Management of patients presenting to GPs with new-onset fatigue is often unclear because it is associated with a range of underlying conditions including, very rarely, cancer. No study has yet quantified the risk of multiple diseases in patients with fatigue in primary care. This study found that out of 237 diseases studied, depression; respiratory tract infections; insomnia and sleep disturbances; and hypo/hyperthyroidism (women only) were most strongly associated with new-onset fatigue presentation. Cancer was the disease with the fourth highest excess risk in men aged 80 years with fatigue, and the thirteenth highest in women aged 80 years with fatigue. Overall, results suggest doctors should prioritise cancer investigation in older men (aged ≥70 years) with fatigue, but not in women at any age, if based solely on the presence of fatigue. These findings provide GPs with information about risk of a comprehensive range of diagnoses in patients with fatigue, and their relative likelihood, so they can prioritise which tests and referral routes to consider.

### Study cohorts and follow-up

**Figure 1.**
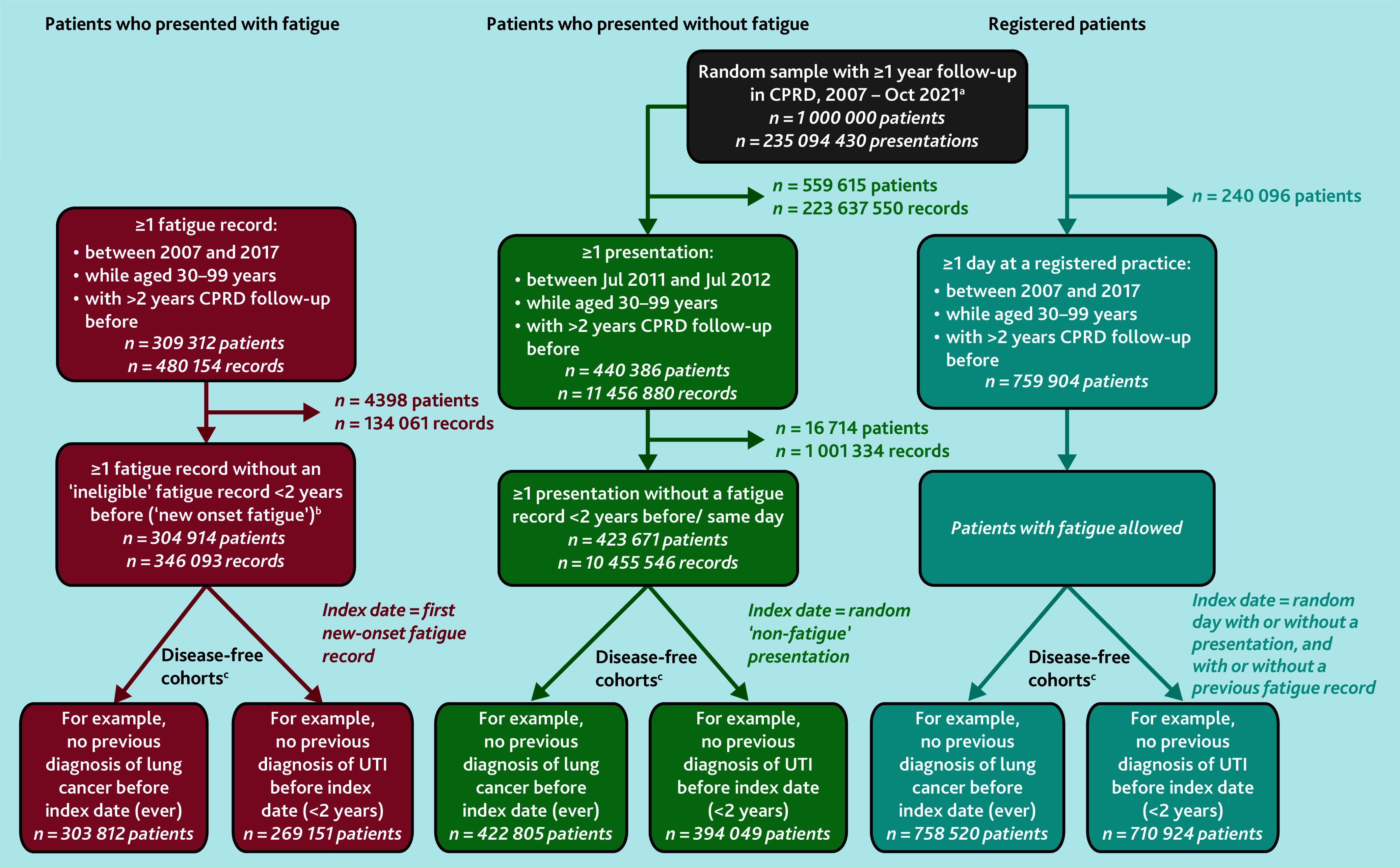
Study cohorts. ^a^Data received from CPRD: patients had to be aged 30–99 years and have ≥1 year CPRD follow-up when selected for the initial random sample cohort. Follow-up in CPRD began after the patient was registered to a CPRD practice and the practice’s records were ‘up to standard’ for research, that is, the date from which the practice offered continuous service with no gaps in the recording of patient deaths or transfers. Follow-up ended when the patient left the practice or died (if applicable), and when data were last collected from the practice. ^b^‘New-onset’ fatigue: a small group of patients were excluded who had a prior ‘ineligible’ record of fatigue (for example, before the patient was aged 30 years) recorded shortly (<2 years) before their first ‘eligible’ record that met inclusion criteria. This ensured that when a patient entered the study with their ‘first’ fatigue record, it was truly new onset and did not begin midway through a series of consultations for fatigue. ^c^‘Disease-free’ cohorts: an individual patient could feature in >1 ‘disease-free’ cohort and be counted as having the outcome for >1 disease. Illustrative disease-free cohorts are shown for the 2-year lookback period for previous diagnoses of selected infections, and the lifetime lookback period for all other diseases. Sample sizes for all disease-free cohorts are included in Supplementary Table S6. CPRD = Clinical Practice Research Datalink. UTI = urinary tract infection.

The main cohort of interest included patients presenting to primary care with ‘new-onset’ fatigue between 1 January 2007 and 31 December 2017, identified from CPRD using Read (version 2) codes developed by the University of Exeter (see Supplementary Table S1). Exclusion criteria are detailed in Figure 1, Supplementary Information S1, and a previous publication.^6^ Follow-up began with the patient’s first eligible record of fatigue. Only patients aged 30–99 years with ≥2 years’ ‘up-to-research-standard’ CPRD data before the index date were included.

To illuminate the degree to which observed disease risks among patients who presented with fatigue (the fatigue group) were related specifically to fatigue, risk was examined in a random sample of ‘patients who presented without fatigue’ (the non-fatigue group) with ≥1 consultation during 2011–2012 without fatigue recorded in the previous 2 years, of which one was randomly chosen as the index date.

Disease risk was further contextualised against that in a random sample of ‘registered patients’, designed to approximate the general population, as disease risk in both the fatigue group and the non-fatigue group could reflect morbidity differences and increased disease severity in patients who present to primary care.^11^^,^^12^ A random index date was chosen from 2007 to 2017, ignoring whether patients consulted or had fatigue recorded (Figure 1).

Follow-up ended at the earliest of: 12 months following the index date, or the first diagnosis of the disease of interest. Twelve months was a compromise to allow adequate follow-up of diseases that may generally take a long time to diagnose (for example, Parkinson’s disease), and the previously evidenced 9-month period of excess cancer risk following fatigue presentation.^6^ A supplementary analysis was included demonstrating variation in excess risk by follow-up time (see Supplementary Figure S1).

Patients who died were not censored during the 12 month follow-up period. They remained in the denominator of patients ‘at risk’, to generate estimates relevant to GPs’ risk assessment at index presentation.^13^ Patients were not censored if they left a CPRD practice, as changing GP practice is not random^14^ and could introduce selection bias.

### Outcomes

In total, 237 conditions were examined relevant to patients aged 30–99 years that could feasibly represent incident disease (see Supplementary Information S1 and Supplementary Table S2), using phenotypes developed by Kuan *et al*,^15^ supplemented with other fatigue-related conditions (see Supplementary Table S3). Data source combinations provided by published phenotypes were used; for most diseases, primary care (CPRD) and secondary care (HES APC) data were combined. For malignant cancers only ‘gold standard’ cancer registry data were used, as CPRD and HES APC may capture ‘false positive’ cases.^16^ Final code lists are available in Supplementary Table S4.

Incident disease risk was estimated in separate cohorts with no previous diagnosis of each disease, as per similar studies.^17^^–^^20^ This meant associations between fatigue and each disease were examined separately, so that each disease risk was agnostic of the risk of other diseases diagnosed during study follow-up. Therefore, a single patient could be represented in multiple ‘disease-free’ cohorts, and a patient in one ‘disease-free’ cohort could also have a previous or subsequent diagnosis of another disease.

Some codes were categorised by Kuan *et al* under >1 disease.^15^ The HES APC phenotypes contained the most duplicates; of the International Classification of Diseases, 10th Revision (ICD-10) codes, 299 were categorised under ≥2 diseases.

For each disease outcome studied, patients who had a previous diagnosis of that disease were excluded from analysis. For most diseases, patients who ever had that disease before their index date were excluded. For some infections, only patients with a diagnosis in the last 2 years were excluded (see Supplementary Table S3). In a sensitivity analysis, the impact of including all patients was examined, regardless of previous diagnoses.

### Statistical analysis

The risk of each disease within 12 months following the index date was calculated. Poisson regression models were used, stratified by sex, with age modelled as a continuous variable using natural cubic splines. Modelled disease risk at selected ages were produced. Robust standard errors were used to account for possible overdispersion. For patients aged >90 years, model fit was generally suboptimal (see Supplementary Information S1), so estimates are not shown. Eighteen impossible disease–sex combinations were excluded from analysis (12 in men and six in women; see Supplementary Table S3). To ensure sufficient precision, diseases with <100 people diagnosed who presented with fatigue in each sex strata were excluded.

Diseases with the greatest absolute excess risk (AER) in the fatigue group compared with the non-fatigue group were identified, after standardising estimates to the age distribution of patients who presented with fatigue. Statistically significant differences were identified where 95% confidence intervals (CIs) around respective disease probabilities did not overlap, which is a conservative approach.^21^ There was no adjustment for multiple testing.

Data management was conducted in MySQL Workbench (version 6.1) and Stata (version 17), and statistical analysis was conducted in R (version 4.1.2). All analytical code is available at https://github.com/rmjlrwh/FatigueRiskMap, and an interactive graphic is available at https://becky-white.shinyapps.io/fatigue_risk_map. The Strengthening the Reporting of Observational studies in Epidemiology (STROBE) guidelines for cohort studies were followed (see Supplementary Table S5).

## Results

### Study cohorts

There were 304 914 patients who had ≥1 eligible ‘new-onset’ fatigue presentation in CPRD between 2007 and 2017. Of the 1 million random sample from CPRD, 423 671 patients had ≥1 eligible non-fatigue presentation between 2011 and 2012, without a fatigue presentation in the previous 2 years. Additionally, 759 904 eligible ‘registered patients’ were identified who had ≥2 years follow-up between 2007 and 2017 (Figure 1).

After excluding patients with previous diagnoses, sex-specific disease-free cohorts ranged from 69 636 men who presented with fatigue without previous hypertension (69.03% of men who presented with fatigue; *N* = 100 881) to 384 098 women who presented with fatigue without previous septicaemia (99.99% of registered women; *N* = 384 105) (see Supplementary Table S6).

In total, 66.91% (*n* = 204 033/304 914) of the fatigue group were women, a higher proportion than in the non-fatigue group (53.70%; *n* = 227 497/423 671) or registered patients (50.55%; *n* = 384 105/759 904) (Table 1). Among men, patients who presented with fatigue tended to be older than patients who presented without fatigue or registered patients, whereas among women the patients who presented with fatigue were slightly younger than patients who presented without fatigue, or registered patients. The fatigue group were most likely to have a previous or subsequent diagnosis of one of the disease groups, followed by the non-fatigue group, and then registered patients.

**Table 1.** Demographic characteristics of study cohorts^a^

**Characteristic**	**Men**						**Women**					

	**Fatigue group**		**Non-fatigue group**		**Registered patients**		**Fatigue group**		**Non-fatigue group**		**Registered patients**	

	***n***	**%**	***n***	**%**	***n***	**%**	***n***	**%**	***n***	**%**	***n***	**%**
**Age group, years**												
30–39	13 313	13.20	32 142	16.38	102 064	27.16	42 246	20.71	40 415	17.77	99 980	26.03
40–49	19 285	19.12	44 183	22.52	87 195	23.20	47 523	23.29	50 312	22.12	80 084	20.85
50–59	20 142	19.97	40 463	20.63	69 985	18.62	36 931	18.10	43 472	19.11	66 349	17.27
60–69	19 534	19.36	40 347	20.57	57 440	15.28	28 867	14.15	41 641	18.30	57 259	14.91
70–79	16 838	16.69	25 245	12.87	36 743	9.78	26 477	12.98	28 725	12.63	41 586	10.83
80–89	10 336	10.25	11 971	6.10	19 245	5.12	18 235	8.94	18 307	8.05	30 248	7.87
≥90	1433	1.42	1823	0.93	3127	0.83	3754	1.84	4625	2.03	8599	2.24

**Age, years, median**	58	—	55	—	49	—	52	—	54	—	51	—

**Previous diagnoses**												
Any disease studied	92 502	91.69	164 835	84.02	274 199	72.96	193 244	94.71	203 744	89.56	325 963	84.86
Benign neoplasm/CIN	5260	5.21	7169	3.65	10 220	2.72	22 544	11.05	21 637	9.51	31 371	8.17
Cancers	8752	8.68	9772	4.98	14 788	3.94	15 665	7.68	16 307	7.17	25 013	6.51
Diseases of the cardiovascular system	38 437	38.10	55 499	28.29	78 301	20.84	52 924	25.94	55 784	24.52	80 927	21.07
Diseases of the circulatory system	16 880	16.73	19 354	9.87	28 915	7.69	20 313	9.96	17 593	7.73	27 097	7.05
Diseases of the digestive system	39 157	38.82	56 313	28.71	85 005	22.62	73 033	35.79	63 521	27.92	93 280	24.29
Diseases of the ear	14 988	14.86	20 906	10.66	29 239	7.78	23 142	11.34	21 553	9.47	30 053	7.82
Diseases of the endocrine system	14 436	14.31	19 354	9.87	26 559	7.07	37 353	18.31	32 349	14.22	48 247	12.56
Diseases of the eye	16 528	16.38	23 093	11.77	32 606	8.68	29 728	14.57	29 748	13.08	43 781	11.40
Diseases of the genitourinary system	36 543	36.22	50 257	25.62	72 800	19.37	79 463	38.95	74 018	32.54	108 916	28.36
Diseases of the respiratory system	32 951	32.66	48 939	24.95	77 683	20.67	67 999	33.33	61 957	27.23	94 202	24.53
Haematological/immunological conditions	10 782	10.69	10 855	5.53	16 058	4.27	35 461	17.38	27 560	12.11	41 397	10.78
Infectious diseases	27 822	27.58	33 386	17.02	56 034	14.91	76 028	37.26	61 226	26.91	102 393	26.66
Mental health disorders	33 057	32.77	43 987	22.42	72 225	19.22	86 770	42.53	71 996	31.65	110 277	28.71
Musculoskeletal conditions	45 417	45.02	70 993	36.19	101 447	27.00	88 438	43.34	87 693	38.55	123 401	32.13
Neurological conditions	13 369	13.25	17 668	9.01	27 578	7.34	38 947	19.09	34 300	15.08	52 930	13.78
Skin conditions	33 884	33.59	54 052	27.55	83 046	22.10	76 676	37.58	75 110	33.02	110 225	28.70

**Subsequent diagnoses**												
Any disease studied	64 018	63.46	78 945	40.24	117 421	31.25	131 853	64.62	104 520	45.94	157 894	41.11
Benign neoplasm/CIN	1390	1.38	1529	0.78	2150	0.57	3958	1.94	2854	1.25	4308	1.12
Cancers	2680	2.66	1974	1.01	3735	0.99	2998	1.47	2159	0.95	3952	1.03
Diseases of the cardiovascular system	18 651	18.49	17 787	9.07	27 465	7.31	22 657	11.10	17 478	7.68	27 637	7.20
Diseases of the circulatory system	6503	6.45	5219	2.66	8389	2.23	7223	3.54	4794	2.11	7954	2.07
Diseases of the digestive system	9479	9.40	9928	5.06	14 532	3.87	16 164	7.92	10 918	4.80	15 894	4.14
Diseases of the ear	2537	2.51	2851	1.45	4113	1.09	4037	1.98	2917	1.28	4305	1.12
Diseases of the endocrine system	5088	5.04	3975	2.03	5734	1.53	14 731	7.22	8303	3.65	12 771	3.32
Diseases of the eye	4722	4.68	5967	3.04	8539	2.27	7507	3.68	6529	2.87	9850	2.56
Diseases of the genitourinary system	12 745	12.63	11 500	5.86	18 636	4.96	20 761	10.18	13 722	6.03	22 097	5.75
Diseases of the respiratory system	12 527	12.42	13 139	6.70	19 498	5.19	20 867	10.23	15 905	6.99	23 597	6.14
Haematological/immunological conditions	5295	5.25	2863	1.46	4307	1.15	12 695	6.22	4906	2.16	8034	2.09
Infectious diseases	18 833	18.67	20 292	10.34	30 244	8.05	49 212	24.12	37 330	16.41	55 341	14.41
Mental health disorders	11 045	10.95	9 036	4.61	14 917	3.97	25 082	12.29	13 478	5.92	21 475	5.59
Musculoskeletal conditions	11 283	11.18	13 870	7.07	19 402	5.16	22 611	11.08	17 476	7.68	25 030	6.52
Neurological conditions	5563	5.51	5592	2.85	8592	2.29	12 354	6.05	9101	4.00	14 837	3.86
Skin conditions	7276	7.21	9176	4.68	13 001	3.46	14 868	7.29	11 359	4.99	16 734	4.36
**Total**	100 881	—	196 174	—	375 799	—	204 033	—	227 497	—	384 105	—

a *Patients diagnosed previously or subsequently with each of the 14 broad disease groups are given as a proportion of all those in the fatigue group, non-fatigue group, or registered patients. The same patient could be diagnosed with ≥1 of the broad disease groups, so the number of patients diagnosed in each broad group do not equal the total number diagnosed with ≥1 of the studied diseases. CIN = cervical intraepithelial neoplasia.*

### Age-adjusted risk

For each of the 237 diseases studied (225 in men, 231 in women), subsequent risk among ‘disease-free’ patients was higher (after age adjustment) in the fatigue group compared with the non-fatigue group for 127 diseases for men (of which, 121 were statistically significant), and 151 diseases for women (130 statistically significant) (see Supplementary Figures S2 and S3; Supplementary Table S6 shows unadjusted estimates).

The other diseases (98 in men, 80 in women) had <100 people diagnosed who presented with fatigue and were excluded from further analysis. Of the 12 diseases and six diseases that were deemed impossible in men and women, respectively, and were excluded from the above analysis, there were four cases of women with erectile dysfunction, and one woman with hyperplasia of prostate.

The risk of all cancers combined in men was 2.59% (95% CI = 2.49 to 2.70) in patients who presented with fatigue; approximately double that in patients who presented without fatigue (1.16%, 95% CI = 1.09 to 1.23), making cancer the disease with the seventh greatest excess risk. In women, cancer risk in patients who presented with fatigue was 1.42% (95% CI = 1.36 to 1.47), which was 0.5 percentage points higher than in patients who presented without fatigue (0.90%, 95% CI = 0.86 to 0.94), and the twenty-first greatest excess risk (Table 2 and Supplementary Table S7).

**Table 2.** Excess risk of cancer and diseases with >1% AER in men or women who presented with fatigue compared with patients who did not present with fatigue, ranked by AER

**Disease**	**Overall absolute risk in the fatigue group, % (95% CI)**	**Overall absolute risk^a^ in the non-fatigue group, % (95% CI)**	**Overall AER^a^ in the fatigue group versus non-fatigue group, %**	**Rank of overall AER^a^**	**Included in current guidelines^b^**
**Men**					
Depression	3.21 (3.09 to 3.33)	0.83 (0.77 to 0.9)	2.38	1	Yes
LowerRTIs	5.55 (5.40 to 5.70)	3.26 (3.15 to 3.38)	2.28	2	Yes
Hypertension	5.09 (4.93 to 5.26)	2.91 (2.79 to 3.04)	2.18	3	No
Insomnia and sleep disturbances	2.55 (2.45 to 2.66)	0.70 (0.64 to 0.76)	1.85	4	Yes
Ear and upper RTIs	4.96 (4.82 to 5.10)	3.16 (3.05 to 3.28)	1.79	5	Yes
UTIs	3.21 (3.10 to 3.32)	1.71 (1.63 to 1.8)	1.49	6	No
All cancers combined	2.59 (2.49 to 2.70)	1.16 (1.09 to 1.23)	1.43	7	Yes
Other or unspecified infectious organisms	2.56 (2.47 to 2.66)	1.30 (1.23 to 1.37)	1.26	8	Yes
Other anaemias	1.68 (1.6 to 1.76)	0.43 (0.39 to 0.47)	1.25	9	Yes
Erectile dysfunction	2.12 (2.02 to 2.22)	0.93 (0.86 to 0.99)	1.19	10	No
Chronic kidney disease	2.12 (2.02 to 2.21)	0.98 (0.92 to 1.04)	1.14	11	Yes
Anxiety disorders	1.53 (1.45 to 1.62)	0.48 (0.44 to 0.53)	1.05	12	Yes
Diabetes	1.82 (1.73 to 1.91)	0.78 (0.73 to 0.84)	1.04	13	Yes
Connective and soft tissue disorders	2.90 (2.78 to 3.02)	1.88 (1.78 to 1.98)	1.02	14	Yes

**Women**					
Depression	3.64 (3.54 to 3.73)	1.28 (1.22 to 1.33)	2.36	1	Yes
UTIs	6.66 (6.54 to 6.78)	4.42 (4.32 to 4.52)	2.24	2	No
Ear and upper RTIs	6.64 (6.52 to 6.76)	4.54 (4.45 to 4.64)	2.10	3	Yes
Hypo or hyperthyroidism	2.43 (2.36 to 2.5)	0.67 (0.63 to 0.71)	1.76	4	Yes
LowerRTIs	4.79 (4.70 to 4.89)	3.28 (3.19 to 3.36)	1.52	5	Yes
Insomnia and sleep disturbances	2.25 (2.18 to 2.32)	0.80 (0.75 to 0.84)	1.46	6	Yes
Anxietydisorders	2.08 (2.01 to 2.15)	0.97 (0.92 to 1.01)	1.11	7	Yes
Hypertension	3.06 (2.98 to 3.15)	1.96 (1.90 to 2.03)	1.10	8	No
Iron deficiency anaemia	1.48 (1.43 to 1.54)	0.42 (0.39 to 0.44)	1.07	9	Yes
Other anaemias	1.52 (1.47 to 1.58)	0.48 (0.45 to 0.51)	1.05	10	Yes
Next 10 diseases with <1% AER not shown	—	—	—	—	—
All cancers combined^c^	1.42 (1.36 to 1.47)	0.90 (0.86 to 0.94)	0.52	21	Yes

a *Adjusting for age differences between the fatigue group and non-fatigue group.*

b *Diseases included in current UK diagnostic guidance for fatigue published by either the National Institute for Health and Care Excellence or in BMJ Best Practice.^4^^,^^5^*

c *Cancer is shown for women despite not having an AER >1%. AER = absolute excess risk. RTI = respiratory tract infection. UTI = urinary tract infection.*

Of diseases already included in UK diagnostic guidelines for fatigue, those with the greatest excess risk in the fatigue group were depression, lower and upper respiratory tract infections (RTIs), insomnia and sleep disturbances, hypo/hyperthyroidism (women), and cancer (men). Some diseases with >1% excess risk are not currently listed in guidelines (for example, hypertension) (Table 2).

### Age-specific excess risk

**Figure 2.**
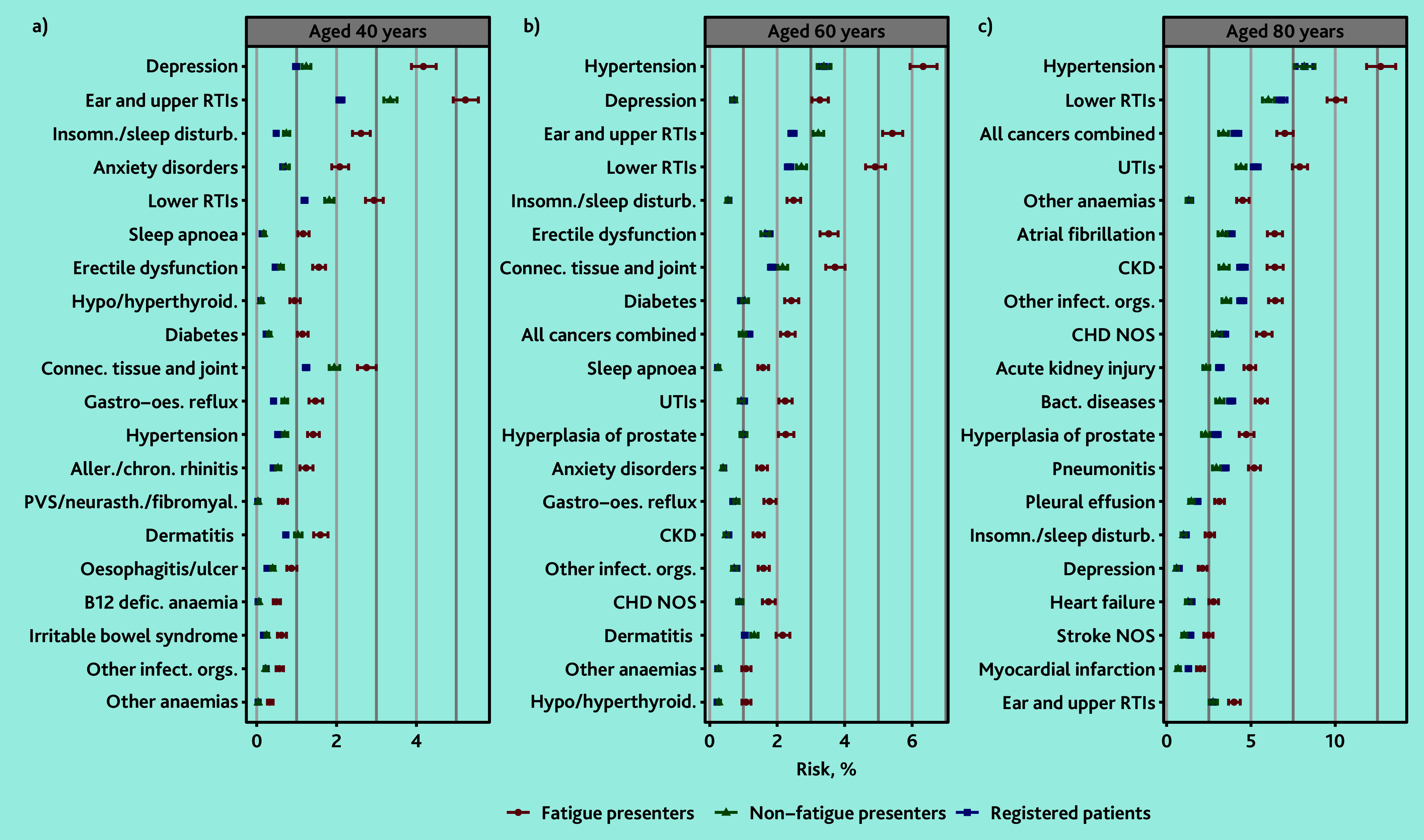
Modelled 1-year disease risk in men at selected ages, by cohort. Top 30 diseases with the greatest absolute excess risk in men who presented with fatigue compared with men who presented without fatigue in those aged a) 40, b) 60, or c) 80 years, ranked by excess risk. Excludes patients with a previous diagnosis of each disease. Aller./chron. rhinitis = allergic and chronic rhinitis. Bact. diseases = Bacterial diseases (excluding tuberculosis). CHD = chronic heart disease. CKD = chronic kidney disease. Connec. tissue and joint = connective and soft tissue disorders. defic. = deficiency. Gastro-oes. reflux = gastro-oesophageal reflux. Hypo/hyperthyroid. = hypo/hyperthyroidism. Insomn./sleep disturb. = insomnia and sleep disturbances. Other infect. orgs. = other or unspecified infectious organisms. NOS = not otherwise specified. PVS/neurasth./fibromyal. = postviral fatigue syndrome, neurasthenia, and fibromyalgia. RTIs = respiratory tract infections. UTIs = urinary tract infections.

The spectrum of diseases with the greatest AER in the fatigue group varied by age. For men at age 40 years, the three diseases with the largest AER in patients who presented with fatigue compared with patients who presented without fatigue were depression (AER 2.94%; 4.18% versus 1.24%), ear and upper RTIs (AER 1.88%; 5.23% versus 3.35%), and insomnia and sleep disturbances (AER 1.87%; 2.61% versus 0.74%). By age 80 years, the largest excess risk was for hypertension (AER 4.53%; 12.69% versus 8.16%), lower RTIs (AER 4.02%; 10.05% versus 6.03%), and all cancers combined (AER 3.65%; 7.01% versus 3.36%) (Figure 2 and Supplementary Table S8).

**Figure 3.**
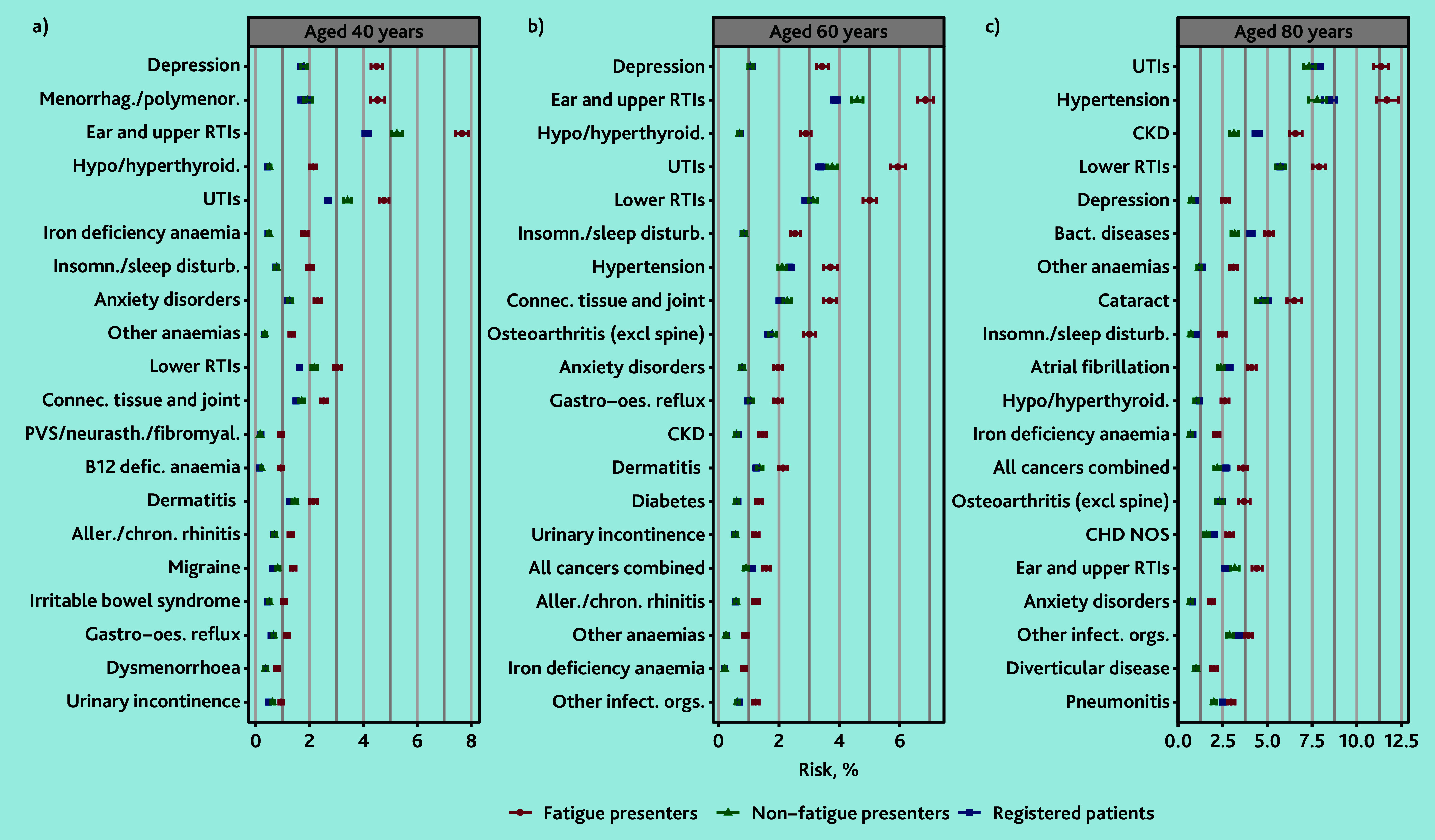
Modelled 1-year disease risk in women at selected ages, by cohort. Top 30 diseases with the greatest absolute excess risk in women who presented with fatigue compared with women who presented without fatigue in those aged a) 40, b) 60, or c) 80 years, ranked by excess risk. Excludes patients with a previous diagnosis of each disease. Aller./chron. rhinitis = allergic and chronic rhinitis. Bact. diseases = bacterial diseases (excluding Tuberculosis). CHD = chronic heart disease. CKD = chronic kidney disease. Connec. tissue and joint = Connective and soft tissue disorders. defic. = deficiency. excl = excluding. Gastro-oes. reflux = gastro-oesophageal reflux. Insomn./sleep disturb. = insomnia and sleep disturbances. Menorrhag./polymenor. = menorrhagia and polymenorrhoea. Other infect. orgs. = other or unspecified infectious organisms. NOS = not otherwise specified. PVS/neurasth./fibromyal. = postviral fatigue syndrome, neurasthenia and fibromyalgia. RTIs = respiratory tract infections. UTIs = urinary tract infections.

For women at age 40 years, diseases with the largest AER were depression (AER 2.69%; 4.49% versus 1.80%), menorrhagia and polymenorrhoea (AER 2.58%; 4.52% versus 1.95%), and ear and upper RTIs (AER 2.42%; 7.65% versus 5.24%). By age 60 years, there was also large excess risk of hypo/hyperthyroidism (AER 2.19%; 2.88% versus 0.70%). By age 80 years, the largest excesses were in urinary tract infections (UTIs) (AER 4.02%; 11.35% versus 7.34%), hypertension (AER 3.91%; 11.68% versus 7.78%), and chronic kidney disease (AER 3.45%; 6.56% versus 3.11%). By age 80 years cancer had the thirteenth greatest excess risk in women (AER 1.45%; 3.64% versus 2.19%). Excess cancer risk increased with age in women but was generally smaller than for men (Figure 3 and Supplementary Table S8).

### Supplementary analyses

Patients with previous diagnoses were excluded. If they were included, risk estimates would be higher in the fatigue group for 209 diseases in men (140 statistically significant) and 222 diseases in women (155 statistically significant), and they would be lower (non-significant) for two diseases (lung and prostate cancer) in men in the fatigue group. Absolute increases were relatively small (<1% higher), except for 17 diseases in men and 17 in women, for which risk was up to 8% higher (see Supplementary Table S6).

For some diseases (for example, lung cancer), risk was concentrated in the first 3–6 months after the index date, whereas for others (for example, depression), risk continued to accumulate at a faster rate in the fatigue group than the non-fatigue group for the entire 12 months’ follow-up (see Supplementary Figure S1).

## Discussion

### Summary

This study quantified the short-term risk of 237 diseases in patients presenting to primary care with new-onset fatigue, including cancer. Over 100 diseases were more common in the fatigue group compared with the non-fatigue group. The largest excesses included depression, RTIs, insomnia and sleep disturbances, and hypo/hyperthyroidism (women only). By age 80 years, cancer had the fourth highest excess risk in men with fatigue, and the thirteen highest in women.

### Strengths and limitations

Primary care EHRs from CPRD were used in this study, which are high quality and broadly representative of the UK population regarding age, sex, and ethnicity.^8^ For most diseases, primary and secondary care data sources were combined using a large-scale disease phenotyping project. By using nationwide hospital episodes data (HES APC), ^22^ it was possible to capture diseases that are more commonly diagnosed in secondary care (for example, via referral to secondary care or emergency presentation). However, this would not capture patients diagnosed for the first time via death certificate only. This could selectively underestimate risk of more serious diseases, particularly in groups at high risk of death (such as older patients). For cancers, national registration data enabled near-complete case ascertainment, although in the UK, a small number of cancers are recorded in CPRD and HES APC and not cancer registry data. Given that cancer registry data are quality assured using various methods, the additional cases recorded in primary and secondary care are likely to be ‘false positive’ cases, where a diagnosis of cancer is suspected initially but excluded subsequently.^16^

To contextualise disease risk in the fatigue group and test the hypothesis that a large portion of disease risk is already present in patients who consult for any reason,^11^ two comparator groups were used in the study. Disease risk was not consistently higher in the non-fatigue group compared with registered patients, suggesting general population and non-symptomatic presenter comparator groups could potentially be used interchangeably in future for some diseases.

The current study aimed to provide a foundational map of disease risk shortly after fatigue presentation to support practical diagnostic management. Therefore, the focus was on risk of specific diseases in patients previously free of these diseases, independently from one another, as per previous studies.^17^^,^^20^^,^^23^ The current study did not examine the co-occurrence of >1 of the studied diseases before or after fatigue presentation.

Relatedly, it was not possible to fully assess mechanisms causing elevated risk of a disease following fatigue presentation, including both direct and indirect causal associations, confounding (for example, because of multimorbidity), or incidental diagnoses (see Supplementary Information S2 and Supplementary Figure S4). The preliminary analysis indicates that compared with patients without fatigue, patients who presented with fatigue are more likely to have a prior diagnosis of ≥1 of the studied diseases, as well as a subsequent diagnosis. The sequencing of diagnoses and trajectories of multimorbidity in patients with fatigue should be examined in future research.

Diagnoses recorded in EHR data shortly after patients first present with fatigue may not reflect the final diagnostic resolution. Some might be initial misdiagnoses that were later revised, so are over-recorded in patients’ EHRs. This could result in overestimating disease risk. However, diseases were ranked by their excess risk in those patients who presented with fatigue compared with patients who presented without fatigue; this should mitigate bias because of mis-recording, assuming that mis-recording is similar in both groups.

Conversely, other diagnoses may have been missed initially, leading to under-recording if they were later diagnosed after a year (the follow-up length used in the current study). Some diseases (for example, depression, as indicated in Supplementary Figure S1), could take >1 year to diagnose. However, the focus of the study was on short-term risk of current but as yet undetected disease. Extending the follow-up period would likely bias estimates of excess risk of current disease by including future disease occurrences unrelated to the fatigue presentation. This applies in particular to diseases that tend to take a very short time to diagnose (for example, cancer).

As full follow-up was available in hospital and cancer registry data, patients were not censored if they left their CPRD practice. This could underestimate risk for diseases that are predominantly recorded in primary care. This is unlikely to be a major problem, as most (85%; *n* = 260 298/304 914) people who presented with fatigue had ≥1 year of follow-up in CPRD, and 92% (*n* = 280 937/304 914) had 6 months.

The current study represents patients with coded fatigue in primary care EHRs, and not people experiencing fatigue in the wider community. People with fatigue but who do not report it might be at lower risk of serious disease. GPs are also more likely to code a symptom rather than record it as free text if they deem it to be serious.^24^ As free text was unavailable, some patients who presented with fatigue may not be included, particularly those with milder fatigue. In addition, the level of ‘under-reporting’ and ‘undercoding’ of fatigue is likely to vary by patient group, as norms about when to seek medical help when experiencing fatigue are likely to be socioculturally determined. For example, such differences may partially explain the elevated cancer risk observed in men compared with women, as women are more likely to report potential cancer symptoms to primary care.^12^ Alternatively, it could reflect a genuine higher prevalence of conditions (other than cancer) associated with fatigue in women than men.^25^

Finally, some diagnoses could have been triggered by another more alarming sign or symptom, although co-occurring ‘alarm’ symptoms for cancer are only recorded in 16% of patients presenting with new-onset fatigue.^7^

### Comparison with existing literature

The current findings reiterate previous studies^6^^,^^7^ that in older men and women presenting with new-onset fatigue the risk of undiagnosed cancer exceeds 3%, the level at which urgent investigation for suspected cancer is recommended in UK guidelines.^26^ The findings extend evidence from previous studies by directly comparing the risk of cancer against other possible diagnoses within a single study. Many of the diseases that had the highest excess risk in those who presented with fatigue (depression, lower and upper RTIs, insomnia and sleep disturbances, hypo/hyperthyroidism [women], and cancer [men]) are already included in UK diagnostic guidelines for fatigue; however, they are listed alongside a long list of other diagnoses, such as Epstein–Barr virus infection, lupus, and cirrhosis,^4^^,^^5^ often in no particular order. The current study offers a way to rank the risk of these differential diagnoses.

To the authors’ knowledge, the only prior attempt to compare the risk of differential diagnoses in patients with fatigue in primary care was a systematic review. This review, similarly to the current study, found that depression was more common in patients presenting with fatigue compared with controls without, but in contrast, cancer was not more common.^27^ However, the review may have masked age-specific associations by summarising evidence for all patients combined, yet the importance of fatigue as a marker of cancer increases with age.

The current study found some diseases (for example, UTIs, hypertension, erectile dysfunction, and cataracts) had >1% excess risk in patients who presented with fatigue but are not currently listed in UK diagnostic guidelines published by the National Institute for Health and Care Excellence (NICE).^5^ They are also not mentioned in key (UK and non-UK based) best practice guidance for fatigue that were reviewed by the current authors,^4^^,^^28^^–^^33^ nor primary studies into fatigue,^1^^–^^3^^,^^25^^,^^27^^,^^34^^–^^36^ with the exception of hypertension in two sources.^28^^,^^35^

Research has identified fatigue as a prodromal symptom of UTIs,^37^ and fatigue is included in UK diagnostic guidance for UTIs published by NICE.^38^ This supports the association found in the current research and suggests UTIs should also be added to UK diagnostic guidelines for fatigue.

As discussed in Supplementary Information S2 and Supplementary Figure S4, diseases with excess risk in patients who present with fatigue may not directly cause fatigue in all cases. Excess risk could be partially explained by indirect causal mechanisms, confounding, or incidental diagnosis. For instance, hypertension is widely considered to be asymptomatic in most people.^39^^,^^40^ Among other mechanisms, indirect causation may explain excess hypertension risk observed in people who present with fatigue, as undiagnosed hypertension can progress to heart failure, which can cause fatigue.^41^

Erectile dysfunction and cataracts are unlikely to directly cause fatigue, although either could indirectly lead to it via their psychosocial impact.^42^^,^^43^ For erectile dysfunction, causation could be reversed, with fatigue directly triggering erectile dysfunction,^44^ or alternatively, both fatigue and erectile dysfunction could be caused by another confounding condition, such as cardiovascular disease,^45^ chronic fatigue syndrome,^46^ depression,^42^ or anxiety.^47^ In practice, large excess risk of a new diagnosis of a particular disease in patients who present with fatigue could still serve as a potential prompt for clinicians to consider that disease, even if indirect causal mechanisms or confounders are involved. However, further research is needed before adding new diseases to diagnostic guidelines for fatigue, particularly if incidental diagnosis could be a factor in their excess incidence following fatigue presentation.

Recent studies have used EHRs to assess cancer risk against a handful of relevant diseases in cohorts of patients presenting with other non-specific symptoms^17^^,^^19^^,^^20^ or abnormal blood test findings.^23^ This information is important but underuses the data and may miss previously unknown associations. The data-driven approach used in the current study is the first, to the authors’ knowledge, to quantify a comprehensive list of diseases.^15^

### Implications for research and practice

The ranking of diseases by excess risk in patients who present with fatigue in the current study could inform UK diagnostic guidelines for fatigue and help GPs prioritise diagnostic testing strategies. The highest likelihood ranks would include depression, lower and upper RTIs, insomnia and sleep disturbances, hypo/hyperthyroidism (women), and cancer (men).

An argument can be made to prioritise investigating cancer in older men (aged ≥70 years) with fatigue, in whom it is relatively likely. In older women, safety-netting for cancer or investigation alongside other diagnoses could be more appropriate. However, other indications from the patient’s medical history, presenting features, or additional tests should also be considered.^4^^,^^5^^,^^30^ The higher likelihood of cancer in men compared with women who presented with fatigue could reflect several factors, including lower cancer risk or higher risk of fatigue-associated non-neoplastic disease in women,^25^ or different help-seeking patterns.^12^

Patients who present with fatigue who have >3% absolute cancer risk may also be at notable risk of other conditions that typically require urgent hospital referral for diagnosis or management. For example, an 80-year-old man with fatigue has a 7% risk of cancer, 5% risk of pneumonitis, and 5% risk of acute kidney injury. When serious disease is suspected, GP access to direct tests^48^ and multidisciplinary referral pathways (for example, rapid diagnostic centres in the UK)^49^ could help expedite diagnosis.

Further research is needed to replicate the current findings in primary care in other (non-UK) healthcare systems, as well as following the COVID-19 pandemic. Associations between recorded fatigue in primary care and undetected disease could vary over time and place according to differences in:

- overall prevalence of different diseases in the population;
- disease-specific aetiology and associated symptoms;
- patient consultation behaviour; and
- doctors’ coding practices of fatigue symptoms and related diagnoses.

The authors are not aware of any studies that have systematically compared the recording of fatigue in primary care across different healthcare systems, nor the associated risk of a range of diagnoses. However, there is evidence that cultural context can determine whether patients attribute chronic fatigue to physical or psychosocial causes,^50^ and some sociodemographic groups are more likely to present with fatigue than others.^51^ This suggests that how often, and which, patients present with the symptom in primary care likely varies by country and so also its associations with different diseases.

In addition, trends in incidence and recording of particular diseases may vary by country. For instance, the recording of chronic fatigue syndrome in the UK has increased dramatically over time.^25^ More recently, the proliferation of long COVID might explain a growing proportion of cases of patients with new-onset fatigue in primary care. As the prevalence and recording of long COVID varies by country,^52^ this might reduce the positive predictive value of fatigue for other diseases in some countries more than others.

In conclusion, the current study systematically examines risk of a wide range of diseases soon after fatigue presentation, demonstrating the potential of EHRs to inform diagnostic guidelines for patients with non-specific symptoms. Age-specific findings support recommendations to prioritise cancer investigation in older men with fatigue but not in women.

## Data

The data used in this study were accessed through the CPRD and are subject to protocol approval by ISAC, therefore they cannot be directly shared. Analysis code and phenotypes are available online at: https://github.com/rmjlrwh/FatigueRiskMap.
